# Probiotic *Bifidobacterium bifidum* G9-1 Has a Preventive Effect on the Acceleration of Colonic Permeability and M1 Macrophage Population in Maternally Separated Rats

**DOI:** 10.3390/biomedicines9060641

**Published:** 2021-06-03

**Authors:** Xuan Wang, Hirokazu Fukui, Ying Ran, Xin Xu, Nobuhiko Ebisutani, Takashi Nakanishi, Yoshiki Tanaka, Ayako Maeda, Yutaka Makizaki, Toshihiko Tomita, Tadayuki Oshima, Hiroto Miwa

**Affiliations:** 1 Division of Gastroenterology and Hepatology, Department of Internal Medicine, Hyogo College of Medicine, l-1, Mukogawa, Nishinomiya 663-8501, Japan; xwang31@tmu.edu.cn (X.W.); ranying1988@126.com (Y.R.); onlyxx1984@163.com (X.X.); no-ebisutani@hyo-med.ac.jp (N.E.); ta-nakanishi@hyo-med.ac.jp (T.N.); tomita@hyo-med.ac.jp (T.T.); t-oshima@hyo-med.ac.jp (T.O.); miwahgi@hyo-med.ac.jp (H.M.); 2 Department of Gastroenterology and Hepatology, Tianjin Medical University General Hospital, Anshan Road 154, Heping District, Tianjin 300052, China; 3 R&D Center, Biofermin Pharmaceutical Co., Ltd., 7-3-4 Higashi-machi, Ibukidai, Nishi-ku, Kobe 651-2242, Japan; tanaka_yoshiki@biofermin.co.jp (Y.T.); maeda_ayako@biofermin.co.jp (A.M.); makizaki_yutaka@biofermin.co.jp (Y.M.)

**Keywords:** *Bifidobacterium*, maternal separation, permeability, macrophage, cytokines, tight junction, CD80, probiotics, stress, claudin

## Abstract

Although probiotics may be useful for the treatment of irritable bowel syndrome (IBS), it is unclear how probiotics play a role in colonic mucosal integrity and immunity. Here, we aimed to investigate the effect of *Bifidobacterium bifidum* G9-1 (BBG9-1) on colonic mucosal integrity and macrophage behavior in rats subjected to maternal separation (MS) as a model of IBS. MS pups were individually separated from their mother rats, and a proportion of the MS rats were orally administered BBG9-1. The colonic mucosal permeability was evaluated by Ussing chamber assay. The expression of tight junction proteins and cytokines and the population of CD80-positive cells was examined in the colonic tissues by real-time reverse transcription-polymerase chain reaction (RT-PCR) and immunohistochemistry. Caco2 cells were stimulated with cytokines and the transepithelial electric resistance (TEER) was measured. MS rats showed significantly higher colonic permeability and lower claudin 4 expression in the colonic epithelium relative to controls. The number of CD80-positive macrophages was significantly increased in the colonic mucosa of MS rats, accompanied by the increase of *IL-6* and *IFN-γ* expression. BBG9-1 treatment ameliorated the increase of M1 macrophage and IL-6/IFN-γ expression in the colonic tissue of MS rats. Simultaneously, BBG9-1 treatment improved the enhanced mucosal permeability and the decreased claudin 4 expression in the colon of MS rats. IL-6 and IFN-γ, whose expression is enhanced in the colon of MS rats, significantly decreased TEER in Caco2 cells in vitro. Probiotic BBG9-1 has a preventive effect on the acceleration of colonic permeability and M1 macrophage population in maternally separated rats.

## 1. Introduction

Irritable bowel syndrome (IBS) is a functional disorder characterized by subjective clinical symptoms such as abdominal pain, bloating, and stool irregularities without any structural or organic lesions [1]. The pathophysiology of IBS is very complicated due to its related numerous factors (visceral sensitivity, bowel motility, mucosal immunity, inflammation, or lifestyle, etc.) [2,3]. Interestingly, gastrointestinal inflammation is likely to cause an inflammatory condition in the brain [4], whereas stress alters the homeostatic balance of not only the hypothalamic-pituitary-adrenal axis but also intestinal mucosal integrity [5], suggesting that the brain–gut axis plays a pivotal role in the pathophysiology of IBS. Psychological stress and the gut microbiome are also key players in the pathophysiology of IBS, and indeed, children who suffered from abuse or received long-term antibiotics are at risk for the development of IBS [6,7]. In this context, we recently investigated maternally separated rats that are recognized as an IBS model and found that psychological stress affects not only the gut microbiome profile but also intestinal mucosal permeability [8]. Moreover, we demonstrated that probiotics treatment (BBG9-1, *Bifidobacterium bifidum*) recovered the altered gut microbiome profile toward that in healthy controls and furthermore improved enhanced mucosal permeability [8]. This finding is well consistent with the fact that BBG9-1 has a protective effect on intestinal mucosal integrity by correcting gut flora in various dysbiotic animal models [9,10,11]. Interestingly, we also clarified that BBG9-1 treatment prevents the susceptibility to early life stress, suggesting that BBG9-1 may play a beneficial role not only in mucosal integrity but also in the function of the brain–gut axis. However, it remains unclear how the probiotics treatment improved the psychological stress-induced alterations in intestinal mucosal permeability. Mucosal permeability is well regulated by tight junction proteins in the intestinal epithelium and greatly affected by the behavior of mucosal immune cells [12]. Therefore, in the present study, we investigated the effect of probiotics treatment not only on tight junction protein expression in the intestinal epithelium but also on the behavior of immune cells in the intestinal mucosa.

## 2. Materials and Methods

### 2.1. Maternal Separation and Experimental Design

Maternal separation (MS) was performed according to the protocol described previously [8,13]. In brief, pregnant Sprague-Dawley rats (gestational day 15) were purchased from Charles River Laboratories Japan (Yokohama, Japan). Mother rats and their litters were randomly assigned to the MS protocol or to the control non-separation protocol. MS pups were individually separated from their mother rats for 3 h per day from days 4 to 19 of life, whereas control pups were left in their home cages with their mother rats.

The probiotics BBG9-1 *(Bifidobacterium bifidum* G9-1; Biofermin R&D Center, Kobe, Japan) was cultured and prepared as previously described [8]. During above experimental period, a proportion of the MS rats were orally administered 50 µL of PBS containing 1 × 10^8^ CFU BBG9-1 once daily immediately after removal from their dam, whereas other MS rats and controls were given orally the same amount of PBS alone as placebo [8,9]. Thus, we created three groups as follows: Control group, neither MS nor BBG9-1 treatment; MS group, MS without BBG9-1 treatment; MSB group, MS with BBG9-1 treatment. To avoid a sex-dependent effect on the MS model, only male rats were subjected to analysis of immunohistochemistry, genetic alterations, and mucosal permeability at postnatal days 20, as we previously reported [8]. Finally, six male rats in each group were subjected to those analyses in this study. All experimental procedures were performed according to the approval by the Experimental Animal Care and Use Committee of Biofermin Pharmaceutical Co., Ltd. (ID: 131-004, 1 August 2016).

### 2.2. Real-Time RT-PCR

Total RNA was isolated from the colonic tissues with TRIzol reagent (Invitrogen; Thermo Fisher Scientific, Inc., Waltham, MA, USA). Total RNA (2 µg) was reverse-transcribed using oligo-dT primer (Applied Biosystems, Branchburg, NJ, USA), and real-time reverse transcription-polymerase chain reaction (RT-PCR) was performed using a 7900H Fast Real-Time PCR System (Applied Biosystems) as described previously [14]. The set of primers for rat IL-1β, IL-6, IL-18, IFN-γ, TNF-α, ZO-1, occludin, claudins, and glyceraldehydes-3-phosphate dehydrogenase (GAPDH) were prepared as shown in Table 1. Real-time PCR assays were performed using 100 ng of RNA equivalent cDNA, SYBR-Green Master Mix (Applied Biosystems) and 500 nmol/L gene specific primers. The PCR cycling conditions were 95 °C for 15 s and 60 °C for 60 s. The intensity of the fluorescent dye was determined, and the expression levels of target genes mRNA were normalized to the expression level of *GAPDH* mRNA.

### 2.3. Tissue Specimen and Immunohistochemistry

The colonic tissues were removed, cut open along the longitudinal axis, and fixed in phosphate-buffered 10% formalin. The fixed tissues were embedded in paraffin and cut perpendicularly to the surface at a thickness of 4 μm.

Immunohistochemical staining for ZO-1, occludin, claudin 3, claudin 4, and CD80 was performed with an Envision Kit (Dako, Carpinteria, CA, USA) as previously described [15], using anti-ZO-1 antibody (dilution 1:200; Invitrogen, Camarillo, CA, USA), anti-occludin antibody (dilution 1:100; Invitrogen, Camarillo, CA, USA), anti-claudin3 antibody (dilution 1:200; Invitrogen), anti-claudin4 antibody (dilution 1:25; Invitrogen, Camarillo, CA, USA), and anti-CD80 antibody (dilution 1:300; Bioss, Woburn, MA, USA). The sections were deparaffinized, rehydrated, and treated by microwave heating for 20 min in Antigen Unmasking Solution (Vector Laboratories, Burlingame, CA, USA) as previously described with minor modifications [16]. To quench endogenous peroxidase activity, the sections were preincubated with 0.3% H_2_O_2_ in methanol for 20 min at room temperature. The sections were then incubated with primary antibodies for 60 min at room temperature, washed in PBS, incubated with horseradish peroxidase-conjugated secondary antibody for 30 min, visualized by 3,3′-diaminobenzidine tetrahydrochloride with 0.05% H_2_O_2_ for 3 min and finally counterstained with Mayer’s hematoxylin.

The number of CD80-positive M1 macrophages was evaluated as follows [17]: Five sections were prepared for the colon in each rat. The positive cells in the lamina propria were counted in at least five different visual fields in a 1000 μm stretch of the entire length in well-oriented tissue sections, and the average was calculated in each rat.

### 2.4. Ussing Chamber Assay

As previously mentioned [8], segments of the distal colon cut along the mesenteric border were mounted in Ussing chambers (Physiologic Instruments, San Diego, CA, USA) to expose a tissue area (0.3 cm^2^) to circulating oxygenated Krebs buffer (115 mM NaCl, 1.25 mM CaCl_2_, 1.2 mM MgCl_2_, 2.0 mM KH_2_PO_4_, 25 mM NaHCO_3_) at 37 °C. Then, mannitol (10 mM) and glucose (10 mM) were additionally included in the Krebs buffer for the mucosal and serosal sides, respectively. The short-circuit current (Isc) values were recorded at equilibrium, ~20 min after the tissues had been mounted and expressed as μA/cm^2^ [18].

### 2.5. Measurement of Transepithelial Electrical Resistance

The human intestinal epithelial cells (Caco2 cell line) were cultured in RPMI 1640 medium (Sigma, Saint Louis, MO, USA) with 10% fetal bovine serum (Gibco, Brooklyn, NY, USA) at 37 °C in a humidified atmosphere of 5% CO_2_. Caco2 cells (2 × 10^4^) were seeded on 24-well culture inserts (0.4 μm pore size; Corning, NY, USA) and grown to confluence. Thereafter, the cells were stimulated in the absence or presence of recombinant human IL-6 (R&D Systems, Minneapolis, MN, USA) or IFN-γ (R&D Systems) in the basolateral chamber up to 72 h later.

Electrical resistance across the stratified epithelium was measured using a Millicell-ERS-2 instrument (Millipore, Bedford, MA, USA) with “chopstick” electrodes, as described previously [19,20]. The value obtained from a blank insert was subtracted to give the net resistance, which was then multiplied by the membrane area to give the resistance in area-corrected units (Ω·cm^2^). The values of transepithelial electrical resistance were recorded in a time-dependent manner after stimulation.

### 2.6. Statistical Analysis

All values are presented as mean ± standard error of the mean (SEM). When a data set was either nonparametric or heteroscedastic, statistical significance was determined by the Steel–Dwass test. When a data set was parametric and homoscedastic, it was determined by the Dunnett’s test. Differences were considered to be significant at *p* < 0.05.

## 3. Results

### 3.1. Effect of BBG9-1 on Intestinal Permeability in MS Rats

**Figure 1:**
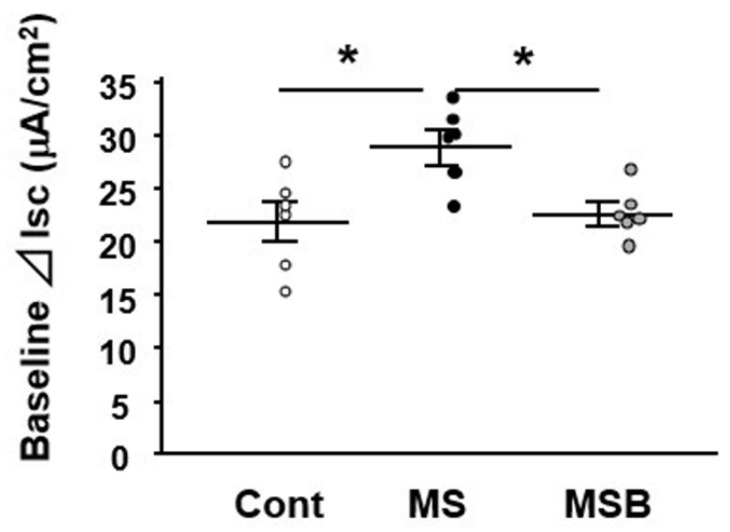
Effect of probiotic BBG9-1 on colonic permeability in MS rats. The macromolecular permeability in the colon tissues was evaluated using the Ussing chamber system. Results are expressed as mean ± SEM. Cont, control (*n* = 6); MS, maternal separation (*n* = 6); MSB, MS with BBG9-1 treatment (*n* = 6). Significant differences between two groups at * *p* < 0.05.

At postnatal day 20, intestinal permeability was measured using the Ussing chamber system ex vivo. The Isc level was significantly greater in the colon of MS rats than in that of controls (Figure 1). However, the increased Isc level in MS rats was attenuated by the treatment with BBG9-1, becoming similar to that in controls.

### 3.2. Effect of BBG9-1 on the Expression of Tight Junction Proteins in the Intestine of MS Rats

**Figure 2:**
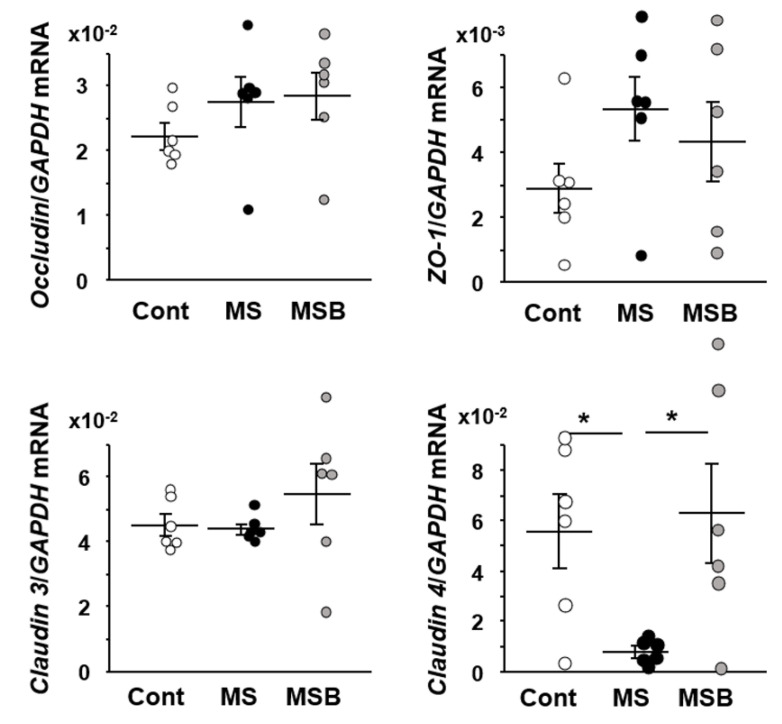
Effect of probiotic BBG9-1 on the mRNA expression of tight junction molecules in the colon of MS rats. Results are expressed as mean ± SEM. Cont, control (*n* = 6); MS, maternal separation (*n* = 6); MSB, MS with BBG9-1 treatment (*n* = 6). Significant differences between two groups at * *p* < 0.05.

We examined the expression of tight junction-associated genes in the colonic tissues (Figure 2). The expression of occuludin, ZO-1, or claudin 3 did not differ among the groups of control, MS, and MS with BBG9-1 treatment. On the other hand, the expression of claudin 4 was significantly decreased in the colon of MS rats relative to controls. Furthermore, its decrease was significantly recovered by the treatment with BBG9-1.

**Figure 3:**
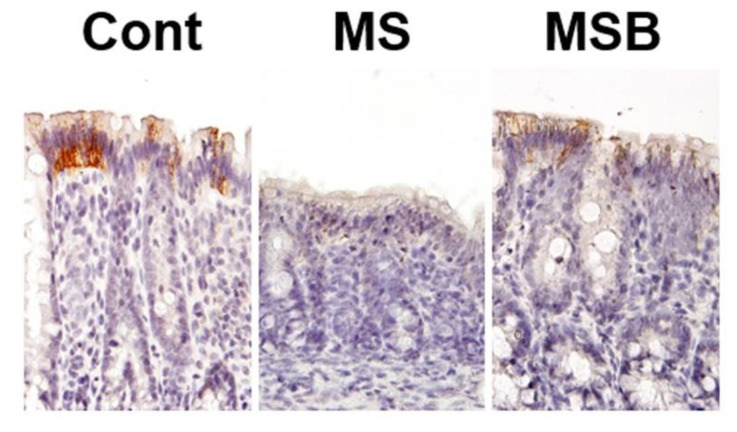
Effect of probiotic BBG9-1 on claudin 4 expression in the colonic mucosa of MS rats. Cont, control; MS, maternal separation; MSB, MS with BBG9-1 treatment. The attenuated immunoreactivity of claudin 4 in MS rats was restored by the treatment with BBG9-1 (magnification ×400).

We next examined the immunohistochemical localization of tight junction proteins in the colonic mucosa of the experimental rats (Figure 3). The immunoreactivity of claudin 4 was detected on the luminal side of the colonic crypts in the control rats. The immunoreactivity of claudin 4 was certainly weaker in MS rats than in controls. However, compatible with the data by real-time PCR, the attenuated claudin 4 expression in MS rats was restored by the treatment with BBG9-1.

### 3.3. Effect of BBG9-1 on the Population of CD80-Positive Cells in the Intestinal Mucosa of MS Rats

**Figure 4:**
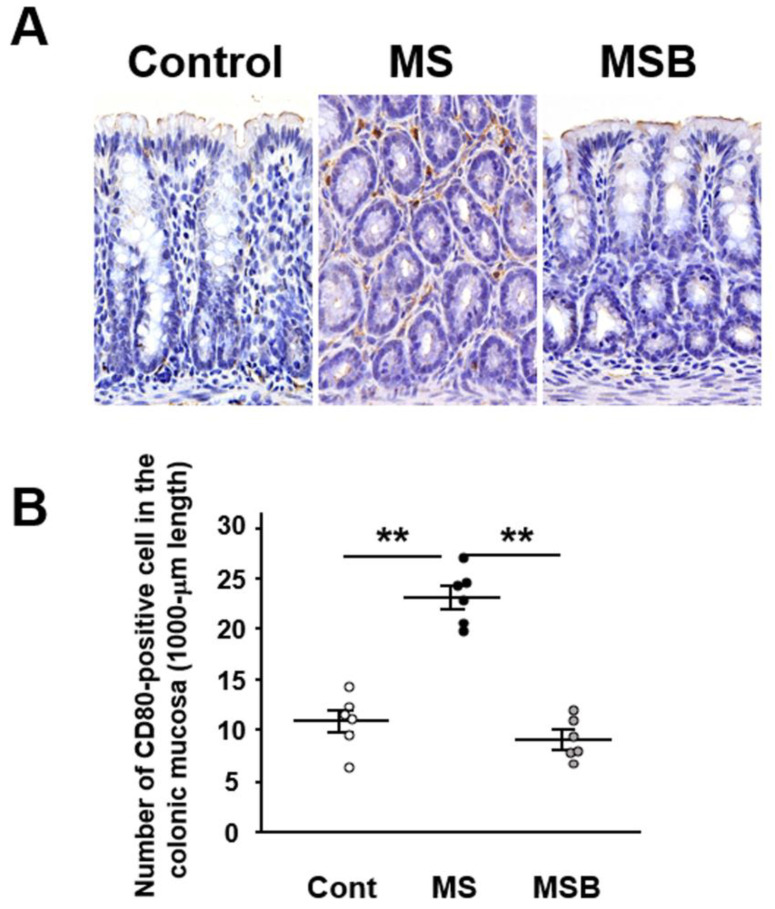
Effect of probiotic BBG9-1 on the population of CD80-positive cells in the colonic mucosa of MS rats. (**A**) Representative immunostaining of CD80 in the colonic mucosa. CD80-positive macrophages are observed in the lamina propria in the colonic mucosa (magnification x400). (**B**) Number of CD80-positive macrophages in the colonic mucosa. Results are expressed as mean ± SEM. Cont, control (*n* = 6); MS, maternal separation (*n* = 6); MSB, MS with BBG9-1 treatment (*n* = 6). Significant differences between two groups at ** *p* < 0.01.

We investigated immunohistochemically the expression of CD80 as a marker of M1 macrophage in the colonic mucosa in the experimental rats. CD80-positive immune cells were detected in the lamina propria of the colonic mucosa (Figure 4A). The number of CD80-positive cells were significantly increased in MS rats compared with controls (Figure 4B). However, the increased population of CD80-positive macrophage was significantly suppressed by the treatment with BBG9-1.

### 3.4. Effect of BBG9-1 on the Expression of Cytokines in the Intestine of MS Rats

**Figure 5:**
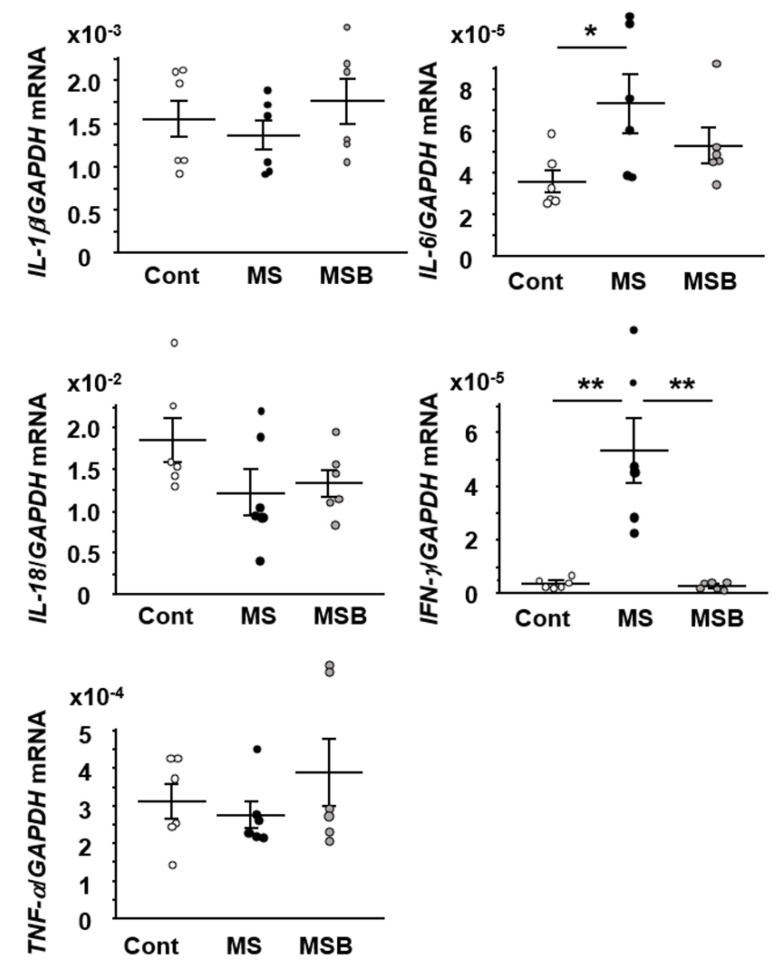
Effect of probiotic BBG9-1 on the mRNA expression of cytokines in the colon of MS rats. Results are expressed as mean ± SEM. Cont, control (*n* = 6); MS, maternal separation (*n* = 6); MSB, MS with BBG9-1 treatment (*n* = 6). Significant differences between two groups at * *p* < 0.05; ** *p* < 0.01.

Since M1 macrophage is increased in the colonic mucosa in MS rats, we investigated the representative gene expression of M1 macrophage-associated cytokines. The expression of IL-6 and IFN-γ was significantly increased in the colon of MS rats relative to the controls (Figure 5). The increased IFN-γ expression in MS rats was significantly attenuated by the treatment with BBG9-1. The increased expression of IL-6 also tended to decrease by BBG9-1 treatment although its alteration did not reach statistical significance.

### 3.5. Effect of Cytokines IL-6 and IFN-γ on Intestinal Permeability In Vitro

**Figure 6:**
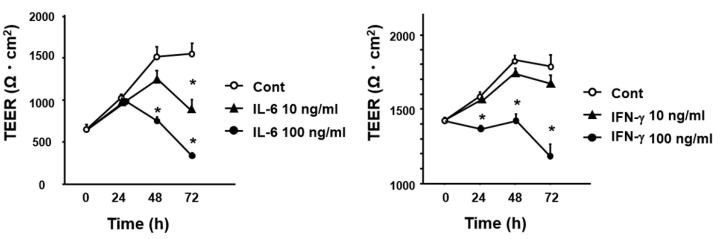
Effect of IL-6 and IFN-γ stimulation on transepithelial electrical resistance (TEER). Caco2 cells were treated and stimulated with recombinant IL-6 (*n* = 3) or IFN-γ (*n* = 3) at indicated concentration as described in Materials and Methods. Significantly lower than the level in untreated controls at the same timepoint; * *p* < 0.05.

As the change patterns of IL-6 and IFN-γ expression were consistent with that of intestinal permeability, we examined the effect of IL-6 and IFN-γ on the permeability of the intestinal epithelial cell layer in vitro. As shown in Figure 6, both of stimulations with IL-6 and IFN-γ significantly reduced the level of transepithelial electrical resistance in Caco2 cell layer.

## 4. Discussion

It has been widely accepted that psychological stress plays a pivotal role in the pathophysiology of IBS. Supporting this consensus, epidemiological studies have reported that subjects who suffered childhood abuse frequently develop IBS [6], and moreover, clinical research has clarified that the severity of anxiety is significantly correlated with the severity of IBS symptom [21]. However, how psychological stress causes disorders such as diarrhea or abdominal pain in IBS patients remains unclear and a crucial concern. It has been proposed that the brain–gut axis plays a pivotal role in the dysmotility or visceral sensitivity in IBS patients [22]. In particular, chronic minimal inflammation has attracted much attention to explain the above hypothesis [23], and moreover, this chronic minimal inflammation may develop due to a leaky gut where the counterbalance among gut microbiome structure, mucosal barrier, and mucosal immune system is disrupted [24]. In this context, we recently demonstrated that the gut microbiome profile is significantly different between IBS patients and healthy subjects [25]. To clarify the pathophysiology of IBS, animal models had been strongly required, and MS rats are widely accepted as a well-established IBS model. Indeed, this animal model exhibits features similar to those of IBS in humans, such as visceral hypersensitivity and enhanced colonic motility in response to acute stress [18,26,27], although the brain functions, behavioral phenotypes, and epigenetic gene expression in the MS model tend to be affected by various conditions including duration, frequency, age, sex, or gut microbiome [28,29]. Of note, we recently reported that psychological stress is likely to affect not only the gut microbiome profile but also intestinal permeability and defecation behavior in IBS model [8]. Based on these data, we further examined the expression of tight junction proteins in the colonic mucosa in the IBS model since those proteins are crucial for regulating the permeability of the colonic epithelium [30]. Subsequently, we found in this study that claudin 4 expression is clearly decreased in the colonic epithelium, being consistent with the enhancement of colonic permeability.

Although we demonstrated the linkage between attenuation of claudin 4 and enhancement of permeability in the colonic tissues, its responsible triggers have to be debated. In this regard, we investigated the alteration of macrophage behavior in the lamina propria located in the basal side of the colonic epithelium because psychological stress is likely to affect intestinal permeability by activating mucosal immune cells [31]. Interestingly, we observed that the population of M1 macrophages is apparently increased in the colonic lamina propria of MS rats, and moreover, that it is associated with cytokines (IL-6 and IFN-γ) expression in the colonic mucosa. These findings suggest that the proinflammatory condition is promoted in the colonic mucosa in rats with psychological stress. Then, we further clarified that both of IL-6 and IFN-γ are able to enhance the permeability of the intestinal epithelial cell layer in vitro. Together, it is tempting to speculate that psychological stress-induced proinflammatory cytokines are likely to enhance the permeability of the colonic mucosa. On the other hand, we are unable to exclude the possibility that the promoted inflammatory condition is a resultant pathophysiology after the enhancement of colonic mucosal permeability. Thus, once mucosal permeability is enhanced due to some cause, the invasion of pathogenic substances (bacteria or antigen, etc.) into the lamina propria would be accelerated [32], leading to the promotion of an inflammatory condition by activated macrophages. Indeed, both mechanisms would occur simultaneously, although it appears impossible to conclude this concern.

We recently confirmed that the gut microbiome profile is different between MS rats and controls [8] and that BBG9-1 treatment prevented the stress-inducible alteration of the gut microbiome profile. Furthermore, it is noteworthy in the present study that BBG9-1 treatment prevented the enhancement of colonic permeability. These findings may suggest that the alteration of the gut microbiome profile at least in part is involved in the regulation of colonic mucosal permeability. On the other hand, one might be concerned if these preventive effects on intestinal barrier integrity are specific in BBG9-1 strains. Since we did not investigate other *Bifidobacterium bifidum* strains in this study, we cannot answer this question. However, it is interesting that not only *Bifidobacterium bifidum* but also other *Bifidobacterium* strains are commonly reported to play a preventive role in intestinal mucosal integrity [33,34,35,36]. Regarding the role of *Bifidobacterium* strains, they facilitate the intestinal colonization of butyrate-producing bacteria [37,38], and the resultant butyrate may be a benefit to protect mucosal integrity [39] and/or to act as an anti-inflammatory factor for mucosal immune cells [40]. In these contexts, although we could not evaluate the short chain fatty acids in the colonic contents, we found that probiotic BBG9-1 had a protective effect on the mucosal barrier and claudin 4 expression in the colon of MS rats. Moreover, it is also interesting that BBG9-1 treatment inhibited the increase of both the colonic M1 macrophage population and proinflammatory cytokines expression in MS rats, suggesting that BBG9-1 may have an anti-inflammatory effect in the colonic mucosa. To support this hypothesis, we should examine whether BBG9-1 treatment ameliorates the disruption of the epithelial cell barrier induced by cytokine stimulation in vitro but this issue remains a limitation to be addressed in a future study. In this context, Nie et al. have shown that *Bifidobacterium* strains suppressed TNF-α-induced overexpression of IL-6 and its associated barrier disruption of Caco2 cells monolayer [41]. Furthermore, Ling et al. demonstrated that *Bifidobacterium* strains enhance the expression of tight junction proteins in Caco2 cells [34]. Together, *Bifidobacterium* strains may ameliorate the disruption of the epithelial cell barrier induced by cytokine stimulation in vitro as well as in vivo.

In summary, we have shown that MS-induced stress develops the elevation of colonic mucosal permeability, the reduction of claudin 4 expression, and the increase of the M1 macrophage population and proinflammatory cytokines expression in the colonic mucosa. However, we know that the interrelationships among psychological stress, enhanced mucosal permeability, and activated proinflammatory condition in the colon still remain to be elucidated. As a limitation of this study, we could not examine the effect of BBG9-1 or other *Bifidobacterium* strains on epithelial cells or immune cells in vitro. However, we have at least clarified that colonic mucosal hyperpermeability and activated inflammatory condition in the IBS model are possible to prevent by treatment with probiotic *Bifidobacterium bifidum*. Therefore, we would like to suggest that the gut microbiome at least plays some role in the interrelationships among psychological stress, enhanced mucosal permeability, and activated proinflammatory condition in the colon and that the gut microbiome is certainly the target for treatment of psychological stress-associated functional gastrointestinal disorders.

## Figures and Tables

**Table 1:** Rat primers for real-time reverse transcription-polymerase chain reaction (RT-PCR) analysis.

Gene	Direction	Primer Sequence
*IL-1β*	Forward Reverse	5′-AATGCCTCGTGCTGTCTGACC-3′ 5′-GGGTGGGTGTGCCGTCTTTC-3′
*IL-6*	Forward Reverse	5′-TCCTACCCCAACTTCCAATGCTC-3′ 5′-TTGGATGGTCTTGGTCCTTAGCC-3′
*IL-18*	Forward Reverse	5′-AAACCCGCCTGTGTTCGA-3′ 5′-TCAGTCTGGTCTGGGATTCGT-3′
*IFN-γ*	Forward Reverse	5′-AGGTGAACAACCCACAGAT-3′ 5′-CTTCTTATTGGCACACTCTCTAC-3′
*TNF-α*	Forward Reverse	5′-TGGCGTGTTCATCCGTTCTCTACC-3′ 5′-CCCGCAATCCAGGCCACTACTT-3′
*ZO-1*	Forward Reverse	5′-GGAAACCCGAAACTGATGCTATGG-3′ 5′-AACTGGCTGGCTGTACTGTGAG-3′
*occludin*	Forward Reverse	5′-AGCAACGATAACCTAGAGACA-3′ 5′-TGTCTCTGTTGATCTGAAGTG-3′
*claudin 3*	Forward Reverse	5′-GGGTTGTACGTGGGCTGGGC-3′ 5′-GTGGATCGCGGCGCGGAATA-3′
*claudin 4*	Forward Reverse	5′-GCCAGCAACTATGTGTAAG-3′ 5′-GCCGTTATGAGTTCAATCC-3′
*GAPDH*	Forward Reverse	5′-CTTGGGCTACACTGAGGACC-3′ 5′-CTGTTGCTGTAGCCGTATTC-3′

## Data Availability

The data presented in this study are available on request from the corresponding author.
